# *Caenorhabditis elegans* Neuromuscular Junction: GABA Receptors and Ivermectin Action

**DOI:** 10.1371/journal.pone.0095072

**Published:** 2014-04-17

**Authors:** Guillermina Hernando, Cecilia Bouzat

**Affiliations:** Instituto de Investigaciones Bioquímicas de Bahía Blanca-Universidad Nacional del Sur, Consejo Nacional de Investigaciones Científicas y Técnicas, Bahía Blanca, Buenos Aires, Argentina; University of Sydney, Australia

## Abstract

The prevalence of human and animal helminth infections remains staggeringly high, thus urging the need for concerted efforts towards this area of research. GABA receptors, encoded by the *unc-49* gene, mediate body muscle inhibition in *Caenorhabditis elegans* and parasitic nematodes and are targets of anthelmintic drugs. Thus, the characterization of nematode GABA receptors provides a foundation for rational anti-parasitic drug design. We therefore explored UNC-49 channels from *C. elegans* muscle cultured cells of the first larval stage at the electrophysiological and behavioral levels. Whole-cell recordings reveal that GABA, muscimol and the anthelmintic piperazine elicit macroscopic currents from UNC-49 receptors that decay in their sustained presence, indicating full desensitization. Single-channel recordings show that all drugs elicit openings of ∼2.5 pA (+100 mV), which appear either as brief isolated events or in short bursts. The comparison of the lowest concentration required for detectable channel opening, the frequency of openings and the amplitude of macroscopic currents suggest that piperazine is the least efficacious of the three drugs. Macroscopic and single-channel GABA-activated currents are profoundly and apparently irreversibly inhibited by ivermectin. To gain further insight into ivermectin action at *C. elegans* muscle, we analyzed its effect on single-channel activity of the levamisol-sensitive nicotinic receptor (L-AChR), the excitatory receptor involved in neuromuscular transmission. Ivermectin produces a profound inhibition of the frequency of channel opening without significant changes in channel properties. By revealing that ivermectin inhibits *C. elegans* muscle GABA and L-AChR receptors, our study adds two receptors to the already known ivermectin targets, thus contributing to the elucidation of its pleiotropic effects. Behavioral assays in worms show that ivermectin potentiates piperazine-induced paralysis, thus suggesting that their combination is a good strategy to overcome the increasing resistance of parasites, an issue of global concern for human and animal health.

## Introduction

GABA-gated chloride channels, members of the Cys-loop receptor family, play an important inhibitory role in the nervous system of vertebrates [1] and invertebrates [2]. Defective GABA neurotransmission causes neurological disorders and GABA_A_ receptors are targets for therapeutically important drugs, including benzodiazepines, barbiturates, and anesthetics [1], [3], [4]. In invertebrates, GABA receptors are important targets for insecticides and nematicides [5]. GABA receptors are homo- or hetero-pentameric proteins composed of an extracellular domain that carries the agonist binding sites and a transmembrane region that forms the ion pore [6]–[8]. The presence of a large number of GABA subunits results in the formation of a wide variety of functionally and pharmacologically different receptor subtypes [1].

In *Caenorhabditis elegans,* body wall muscles receive innervations from both cholinergic (excitatory) and GABAergic (inhibitory) motor neurons. Acetylcholine released from motor neurons stimulates muscle contraction on one side of the body, and simultaneously activates an inhibitory motor neuron that projects to the opposite side of the body to release GABA. Thus, GABA elicits muscle relaxation, facilitating the smooth body bend necessary for coordinated locomotion [2]. Muscle cells contain one GABA and two types of acetylcholine receptors (AChR), the levamisole-sensitive (L-AChR) and the nicotine-sensitive AChR (N-AChR). The *C. elegans* muscle GABA receptor is encoded by the *unc-49* gene, which is translated into three subunits: UNC-49A, UNC-49B, and UNC-49C. In adult *C. elegans,* the GABA receptor has been shown to be composed of UNC-49B and C subunits [9]. The UNC-49B subunit confers synaptic localization and allows channel activation whereas UNC-49C is a non essential modulatory subunit that co-assembles with UNC-49B. The *unc-49* null mutant exhibits the “shrinker” phenotype, owing to hypercontraction of the body wall muscles on both sides of the body. The worms become resistant to muscimol, which is a full agonist of vertebrate GABA_A_ receptors. This drug relaxes all body wall muscles and causes lengthening of adult worms [10], [11].

The *unc-49* gene is also present in parasitic nematodes, including *Haemonchus contortus* [12], *Brugia malayi* and *Trichinella spiralis* [13], *Ascaris suum* and *Loa loa* [5], and GABA receptors are targets of widely used anthelmintic agents. Within these agents, piperazine (PZE) is the active constituent of over-the-counter remedies for threadworm infection in children. Its mode of action has been primarily studied in *A. suum,* where its GABA-mimetic action causes a flaccid, reversible paralysis of body wall muscle [14].

Ivermectin (IVM) has been used successfully to treat billions of humans, livestock and pets. It activates glutamate-activated chloride channels (GluClR) in nematodes and also activates and modulates a great variety of vertebrate and invertebrate Cys-loop receptors, including GABA receptors [15]. Understanding the molecular basis of IVM wide spectrum of action may be important for the development of novel anthelmintic agents.

UNC-49 receptors share significant structural and pharmacological overlap with mammalian GABA_A_ receptors in some aspects and differ greatly in others. These differences could be exploited in parasitic drug design. However, information about functional properties of nematode GABA receptors is still scarce. We therefore here explored macroscopic and single-channel properties of GABA receptors from *C. elegans* L1 muscle cells, determined how they are activated and modulated by anthelmintic agents, and evaluated the behavioral effects of these agents in the worm.

The characterization of GABA receptors in a genetically tractable organism and model of parasitic nematodes provides new avenues of exploration for selective drugs as well as for defining structural determinants of activation and modulation in the Cys-loop receptor family.

## Materials and Methods

### Caenorhabditis elegans strains

Nematode strains used: N2: Bristol wild-type; PD4251: *ccIs4251;dpy-20(e1282)* which produces GFP in all body wall muscles and vulval muscles; EG1653: *oxIs22* [*unc-49p*::*unc-49*::GFP + *lin-15*(+)] psoralen integration of *oxEx129* [*unc-49Bp*(long)::GFP + *lin-15*(+)], which expresses GFP associated to UNC-49R [16]; and the null mutants CB407: *unc-49(e407)* in which the UNC-49B essential subunit is disrupted [16]; DA1316: *avr-14(ad1302);avr-15(ad1051);glc-1(pk54)* in which a simultaneous mutation of three genes, *avr-14*, *avr-15*, and *glc-1*, encoding glutamate-gated chloride channel (GluCl) alpha-type subunits confers high-level resistance to the antiparasitic drug ivermectin [17]. All nematodes strains were obtained from the *Caenorhabditis* Genetic Center, which is funded by the NIH National Center for Research Resources (NCRR). Nematode strains were maintained at 18–25°C using freshly prepared Nematode Growth Medium (NGM) petri dishes that have been spread with *Escherichia coli* (OP50) as a source of food [18], [19].

### Isolation and culture of *C. elegans* muscle cells

Cells were isolated and cultured as described before [18]–[20]. Briefly, adult nematodes were exposed to an alkaline hypochlorite solution and eggs were treated with 1 unit/ml chitinase. The dissociated embryo cells were filtered and placed on glass coverslips coated with poly-O-Ornithine. Cultures were maintained at 22–24°C in a humidified incubator in L-15 medium containing 10% fetal bovine serum. Complete differentiation to the various cell types that comprise the newly hatched Larva 1 (L1) was observed within 24 h as described by Christensen *et al*. [21]. Electrophysiological experiments were performed 1–5 days after cell isolation.

### Patch-clamp recordings

Single-channel currents were recorded in the cell-attached patch configuration at 20°C [18], [19]. The bath and pipette solutions contained 140 mM NaCl, 3 mM CaCl_2_, 5 mM KCl, 5 mM MgCl_2_, 11 mM glucose, 5 mM HEPES (pH 7.4) for UNC-49 receptors and 142 mM KCl, 5.4 mM NaCl, 1.8 mM CaCl_2_, 1.7 mM MgCl_2_, and 10 mM HEPES (pH 7.4) for L-AChRs. Drugs were obtained from Sigma-Aldrich Co. The stock solution for ivermectin (IVM) was prepared in dimethyl sulphoxide (DMSO) and the final DMSO concentration used in all assays was lower than 0.1%. Single-channel currents were recorded using an Axopatch 200 B patch-clamp amplifier (Molecular Devices), digitized at 5 µs intervals, and detected by the half-amplitude threshold criterion using the TAC 4.0.10 program (Bruxton Corporation). Open- and closed-time histograms were plotted using a logarithmic abscissa and a square root ordinate and fitted to the sum of exponential functions by maximum likelihood using TACFit (Bruxton Corporation).

To recognize bursts and quantify their durations, a critical closed time (τ_crit_) was defined as the point of intersection between the briefest and the succeeding component, and openings separated by closings briefer than this time constitute a burst [22]. Typically, τ_crit_ ranged from 0.15 to 0.20 ms. Burst duration histograms were well described by the sum of two exponentials, with the briefest duration component corresponding to isolated events and the longest duration component, to bursts. The burst duration was taken from the duration of the slowest component of the burst duration histogram.

Macroscopic currents were recorded in the whole-cell configuration at 20°C at a holding potential of −70 mV [19]. The pipette solution contained 134 mM KCl, 10 mM EGTA, 1 mM MgCl2, and 10 mM HEPES (pH 7.3). The extracellular solution (ECS) contained 140 mM NaCl, 3 mM CaCl2, 5 mM KCl, 5 mM MgCl2, 11 mM glucose, 5 mM HEPES (pH 7.4). The cell membrane capacitance (Cm) was determined using the software Windows Whole Cell Program (WinWCP; Strathclyde Institute of Pharmacy and Biomedical Sciences, UK) after obtaining the whole-cell configuration. All tested L1 muscle cells exhibited Cm values varying from 2 to 6 pF. The perfusion system consisted of solution reservoirs, manual switching valves, a four channel VC3 valve controller (ALA Scientific Instruments), a solenoid-driven pinch valve, and three tubes (0.3 mm inner diameter) inserted into the culture dish [23], [24]. One tube contained the ECS alone and the others, ECS with drugs. The cell was positioned within several hundred microns of the three output tubes where streams coming from them would intersect. The solution exchange time was estimated by the open pipette method, by stepping the patch held at 0 mV in ECS and then applying a 2000-ms pulse of ECS diluted 1:1 with water. Typical exchange times ranged from 1 to 2 ms. Macroscopic currents were filtered at 5 kHz, digitized at 20 kHz, and analyzed using WinWCP. Each current represents the average from 2–4 individual traces obtained from the same cell, which were aligned with each other at the point where they reached 50% of maximum. Peak currents (Ipeak) correspond to the value obtained by extrapolation of the decay to this point. The rise time of the currents (t20–80) was determined by calculating the time in which the current increased from 20 to 80% of the peak amplitude. Decays were fitted by a double-exponential function:
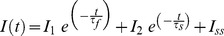
(Equation 1)Where I_ss_ is the steady state current, and τ_f_ and τ_s_, the decay time constants.

### Confocal Microscopy

Cells were grown in cultured dishes from MatTek (glass bottom culture dishes Nº 0 MatTek Corporation, Ashland, MA, U.S.A). Fluorescence images were obtained by laser scanning confocal microscopy (Leica TCS SP2) with a 63X water objective. Images were collected and processed with LCS software (Leica) and Photoshop (Adobe Systems, San Jose, CA). The percentage of green cells was determined by counting cells in ten different fields for each dish (n = 3 dishes). The result is expressed as the mean ± SD from three different cell isolations. Quantitative analysis of GABA receptor distribution was conducted by measuring the intensity profile along the cell on the focal plane of the membrane by using LCS software (Leica). An arbitrary 7.5 µm line was traced through the cell starting and ending it in a background zone and a curve of fluorescence intensity was constructed for each point of the line.

### Motility assays

Assays were performed with young adult hermaphrodites or L1 worms from synchronized plates. **Measurement of drug sensitivity.** Paralysis was determined on agar plates containing the tested drug at room temperature as described before [19]. Body paralysis was followed by visual inspection at the indicated time (every 30–60 minutes) and was defined as the lack of complete body movement in response to prodding. We evaluated different types of nematode paralysis: flaccid that is mediated by inhibitory stimulation, in which worms appear lengthened; spastic paralysis that is mediated by AChR stimulation and worms appear shorter [19], [25], and stationary, in which worms do not show either flaccid or spastic paralysis. Stationary worms retain normal muscular rigidity and the capability of responding to prodding by contracting body wall muscle [26].

#### Thrashing assay

individual young adult *C. elegans* were placed in 200 µl of M9 buffer in the absence or presence of the drug in a 96-well microlitre plate. After 30 minutes in the solution at room temperature, the number of thrashes (bends of the body from one side to the other) were counted during 1 minute [19]. The experiments were repeated 3 times for each condition (10 worms tested each time).

### Statistics

Experimental data are shown as mean ± S.D. Statistical comparisons were done using the Student's *t* test or one-way ANOVA with Bonferroni's multiple comparison post test. A level of *p*<0.05 was considered significant.

## Results

### L1 muscle cells respond to GABAergic agonists

**Figure 1:**
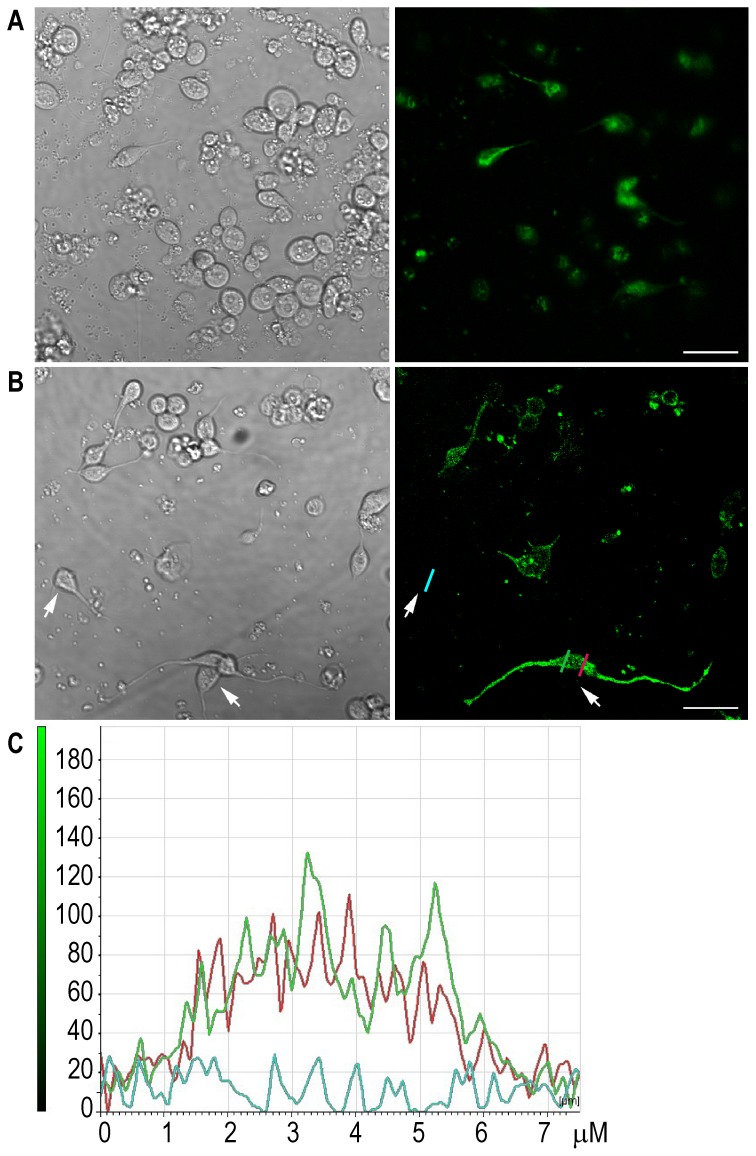
Expression of GABA receptor in *C. elegans* muscle cultured cells. Confocal microscopy of L1 muscle cells in culture from worms expressing GFP in muscle (PD4251 strain, (**A**)) or associated to GABA receptors (EG1653 strain, (**B**)). Left panels correspond to DIC and Right panels correspond to fluorescence microscopy. Bar: 20 µm. (**C**) Fluorescence intensity profile at the membrane level of representative cells shown in panel B, two of which show GFP expression (marked by green and red lines) and the other corresponds to the basal fluorescence (blue line).

Cultured muscle cells corresponding to the Larva 1 stage (L1) can be identified by their spindle-shaped morphology (Fig.1; [21]). In the PD4251 strain, which produces GFP in all body wall and vulval muscles, muscle cells can be easily distinguished by fluorescence microscopy (Fig. 1A, right panel). Confocal microscopy observation of cultures obtained from the EG1653 strain, which expresses GFP associated with GABA receptors [16], shows that 78±10% of cultured cells with spindle-shaped morphology express GABA receptors, which appear to be homogeneously distributed on the cell membrane (Fig. 1B and C).

**Figure 2:**
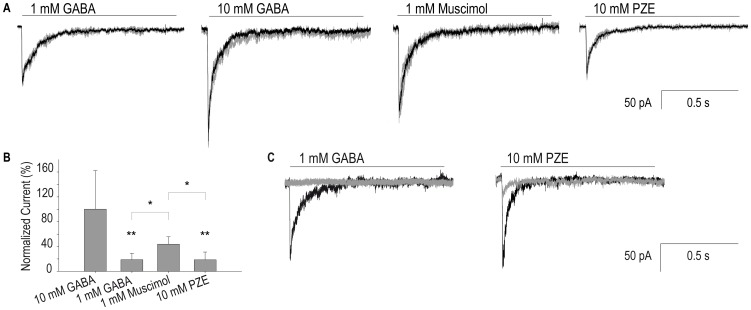
Macroscopic responses of L1 muscle cells to GABAergic agonists. (**A**) Representative whole-cell currents from L1 cultured muscle cells (PD4251 strain) elicited by a 1 s-pulse of GABA, muscimol and piperazine. Pipette potential: −70 mV. Each current corresponds to a different cell and represents the average of at least 2–4 applications of the same agonist per cell. (**B**) Percentage of the peak current normalized to that elicited by 10 mM GABA. ANOVA with Bonfferroni multi comparison post test. (* *p*<0.05, ** *p*<0.01). (**C**) Whole-cell currents activated by 1 mM GABA or 10 mM PZE before (black line) and after pre-exposure to 30 µM IVM during 2 min (grey line).

To verify that L1 muscle cells express functional GABA receptors, we measured macroscopic currents elicited by GABA in the whole-cell configuration at −70 mV pipette potential. In the control PD4251 strain, 80±12% of muscle (green) cells showed responses to 1 mM GABA application whereas no GABA-activated currents were detected from L1 muscle cells derived from *unc-49(e407)* mutant [16], which lacks UNC-49 receptors. 1 mM GABA-elicited currents reach a peak in less than ∼4 ms (t_20-80_  =  4±2 ms, n = 13; Fig. 2A). The mean peak current (I_peak_) is -21.7±11 pA/pF at −70 mV. Currents decay in the continuous presence of GABA to reach steady state current values (I_ss_) close to zero (I_ss_/I_peak_  =  0.05±0.03), indicating full desensitization. Decays are fitted by a double exponential function (τ_fast_  =  19±9 ms and τ_slow_  =  85±40 ms). At 10 mM GABA, peak currents are between 4- and 5-fold larger than at 1 mM (−105.7±65 pA/pF, n = 9; Fig. 2A and B). Despite the variability in current amplitudes among different cells, the difference between 1 and 10 mM GABA is statistically significant (Fig. 2B). Muscimol (1 mM) also elicits GABA-activated currents from L1 muscle cells with a mean peak current of −47.8±13 pA/pF (n = 5), which is statistically different from that of 1 mM GABA-currents (Fig. 2A and B). 1 mM piperazine (PZE) does not elicit detectable currents. However, 10 mM PZE elicits currents in ∼76% of the cells, indicating that PZE acts as an agonist of *C. elegans* UNC-49 receptors. Peak currents are smaller and statistically different to those elicited by 10 mM GABA (I_peak_  =  −20±13 pA/pF, n = 5; Fig. 2B). Increasing PZE concentration to 25 mM does not produce larger currents than those elicited by 10 mM PZE (I_peak_  =  21.7±6 pA/pF n = 3; *p*>0.30), suggesting that this drug does not behave as a full agonist. Unfortunately, an extended pharmacological study in the same cell is not possible because of the seal stability and the resealing of the whole cell. Nevertheless, the results suggest that PZE is less efficacious than GABA and muscimol at *C. elegans* UNC-49 receptors.

Ivermectin (IVM) has been shown to activate and modulate a large number of vertebrate and invertebrate Cys-loop receptors. We therefore explored if it also affects GABA receptors from *C. elegans*. Currents are not elicited by application of IVM, indicating that it is not capable of activating GABA receptors (n = 6 cells). Pre-exposure of L1 cells to 30 µM IVM during 2 min fully inhibits 1 mM GABA-responses (n = 6 cells) and inhibits 90±3% of 10 mM PZE-responses (n = 8 cells, Fig. 2 C). Peak currents cannot be recovered after 5-min wash with buffer alone, suggesting apparent irreversibility. As a control, we verified that GABA-activated currents are not inhibited when cells are pre-exposed 2 minutes to 0.1% DMSO in the absence of IVM (n = 3, peak current >85% of the control peak). Thus, we conclude that IVM acts as an inhibitor of *C. elegans* UNC-49 receptors.

### Single-channel currents of GABA receptors from L1 muscle cells

**Figure 3:**
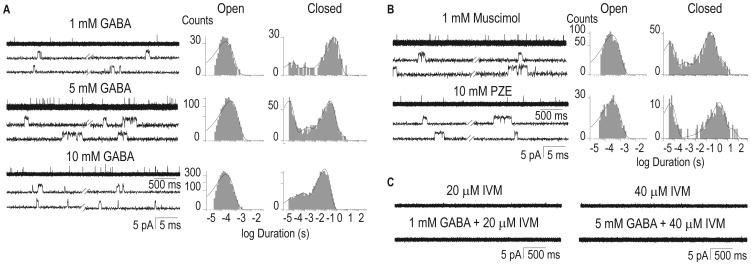
Single-channel currents from GABA receptors recorded from cultured muscle cells. Single channels currents were recorded from muscle cultured cells in the presence of GABA (1, 5 or 10 mM) (**A**) and 1 mM muscimol or 10 mM piperazine (PZE) (**B**). Representative channel traces and open and closed time histograms are shown for each condition. (**C**) Representative traces of single-channel activity in the presence of IVM alone (top traces) or together with 1 mM or 5 mM GABA in the pipette solution (bottom). Channel openings are shown as upward deflections. Pipette potential: +100 mV. Filter: 9 kHz.

To gain further insight of how anthelmintic drugs act on GABA receptors we recorded single-channel currents from cell-attached patches in L1 muscle cells. Single-channel openings are detected at concentrations higher than 0.5 mM although they are very infrequent at this concentration. The proportion of patches that show detectable single-channel activity is very low at all GABA concentrations. At 5 mM GABA, only ∼15% of the patches show detectable openings. Channel openings exhibit single amplitudes of 2.5±0.6 pA at +100 mV pipette potential (Fig. 3A), 2.9±0.3 pA at +140 mV, and 3.5±0.2 pA at +160 mV (n = 6). By the slope of the current-voltage relationship, we estimated a value of 22±0.8 pS (n = 6) for channel conductance.

No opening events were detected in the absence of GABA (n = 30 cells) or in cell-attached patches from the *unc-49(e407)* mutant (n = 10 cells), thus revealing that the observed channel activity is originated from UNC-49 receptors.

At all GABA concentrations (1-10 mM), single-channel activity occurs as isolated opening events and short bursts composed of successive openings (Fig. 3A). Open-time distributions at all GABA concentrations are well described by a single exponential component with mean durations between ∼0.18 and 0.40 ms (Fig. 3A and Table 1). Bursts are readily detected at 5 mM GABA and show a mean duration of 1.1±0.25 ms (n = 5; Fig. 3A). Closed time distributions can be well fitted by three or four exponential components (Fig. 3A). The duration of the main closed component decreases with GABA concentration, indicating the increase in the frequency of openings with agonist concentration (Fig. 3A and Table 1).

**Table 1:** Single-channel properties of GABA receptors and levamisole-sensitive AChRs.

Agonist (mM)	O_1_ (ms) (area)	O_2_ (ms) (area)	C_2_ (ms) (area)	n
**UNC-49 receptors**				
GABA 1	0.26±0.04 (1)	nd	1100±300 (0.85±0.03)	3
GABA 3	0.20±0.03 (1)	nd	220±140 (0.80±0.07)	3
GABA 5	0.39±0.09 (1)	nd	180±70 (0.77±0.17)	9
GABA 10	0.18±0.03 (1)	nd	87±70 (0.85±0.01)	4
Muscimol 1	0.40±0.17 (1)	nd	950±500 (0.57±0.09)	5
PZE 10	0.40±0.11 (1)	nd	1900±400 (0.58±0.14)	5
**L-AChRs**				
ACh 0.05	0.24±0.03 (0.89±0.01)	0.40±0.06 (0.11±0.01)	13.60±6.2 (0.70±0.01)	5
ACh 0.05 + IVM 0.05	0.26±0.07 (0.87±0.08)	0.41±0.16 (0.17±0.04)	48.40±20 (0.58±0.11)	5

Single-channel currents were recorded from L1 muscle cells at a holding potential of +100 mV. GABA channels were recorded in the presence of GABA, muscimol and piperazine in the pipette solution. ACh channels corresponding to L-AChRs were recorded in the presence of ACh with and without IVM in both pipette and dish solutions. The data were obtained from the corresponding histograms and correspond to the average of different recordings (n) for each condition. nd: Non detected.

Single UNC-49 channel activity is also detected in the presence of 1 mM muscimol and 10 mM PZE (Fig. 3B). As expected, the amplitude of opening events is similar to that of GABA-activated channels (2.4±0.8 pA and 2.5±0.6 pA for 1 mM muscimol and 10 mM PZE, respectively, *p*>0.8; Fig. 3B). Mean open times (Table 1) and burst durations (0.94±0.29 ms and 1.2±0.14 ms for 1 mM muscimol and 10 mM PZE, respectively) are not statistically different from those of 5 mM GABA-activated channels (*p*>0.4). The frequency of opening events from recordings in the presence of 10 mM PZE is systematically lower than that of 10 mM GABA (See representative traces in Fig. 3). The reduced frequency is clearly reflected in the closed time histograms by the displacement to longer durations of the main closed component (Fig. 3 and Table 1). This result is in agreement with the smaller macroscopic responses elicited by PZE, again suggesting that this drug is less efficacious than GABA.

Single-channel activity is not detected when IVM (20–40 µM) alone is present in the pipette solution (n = 12 and n = 14 for 20 and 40 µM IVM, respectively), indicating that it is not capable of activating *C. elegans* GABA receptors (Fig. 3). Also, GABA (1, 5 or 7 mM) does not elicit detectable single-channel activity if IVM is present in the pipette solution (n = 24 and n = 28 for 20 and 40 µM IVM, respectively; Fig. 3C). This result reveals that IVM produces fast and apparently irreversible inhibition of GABA receptors.

### Effects of ivermectin on levamisole-sensitive AChRs

**Figure 4:**
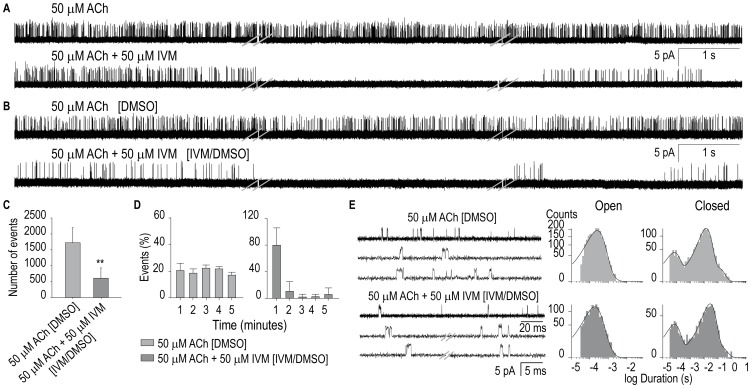
Modulation of L-AChRs of L1 muscle cells by ivermectin. (**A**) Single-channels activated by 50 µM ACh were recorded in the absence (top) and presence of 50 µM IVM in the pipette solution (bottom). Three different traces are shown for each condition and correspond to the recording during the first, third and fifth minute. (**B**) Representative traces of single-channels activated by 50 µM ACh were recorded in the absence (top) and presence of 50 µM IVM in the pipette solution (bottom) from cells previously incubated with 0.1% DMSO (top) or 50 µM IVM in 0.1% DMSO (bottom). In brackets it is indicated the pre-incubation condition (0.1% DMSO for the control and 0.1% DMSO plus 50 µM IVM. (**C**) Comparison of the number of opening events in the first 30 seconds of recording as in (B). ** *p*<0.01 (Student *t*-Test). (**D**) Bar chart showing the percentage of opening events per minute during the first 5-min recording as in (B). The data correspond to five different recordings for each condition. (**E**) Representative traces and open and closed time histograms of channels recorded as explained in Panel B. Traces are shown at two temporal scales. Pipette potential: +100 mV. Filter: 9 kHz.

Since the L-AChR is the main Cys-loop receptor involved in muscle contraction, we evaluated whether it is, as the muscle GABA receptor, modulated by IVM. To this end, we took advantage of our previous characterization of single-channel activity of L-AChR on L1 cells [18], [19]. As shown in Figure 4A, single channels of L-AChRs activated by 50 µM ACh are readily detected from L1 muscle cells. Channel activity shows openings of 3.5±0.3 pA (n = 5) at +100 mV pipette potential that appear isolated or in short bursts (Fig. 4A, top trace). As previously described [18], [19], [27], single-channel activity remains constant during the course of the 15-min recording. In contrast, when 50 µM IVM is present in the pipette solution together with 50 µM ACh, single-channel activity is detected at the beginning of the recording but it dramatically decreases after the first minute; only sporadic and infrequent activation episodes are observed thereafter (Fig. 4A, lower trace). As a control, we verified that 0.1% DMSO does not produce any change in single-channel activity (n = 5).

We also tested the effect of the pre-exposure of cells to 50 µM IVM on single-channel activity. To this end, we incubated the culture dishes for ∼15–20 min with 0.1% DMSO in the absence (Fig. 4B, top trace) or presence (Fig. 4B, lower trace) of 50 µM IVM and recorded ACh-activated single-channel currents as described above. In the first 30 seconds of recording, the number of opening events is significantly reduced in IVM-treated cells and it is statistically different from that of 0.1% DMSO-treated cells (n = 5, *p*<0.01; Fig. 4C). To quantify the decrease of single-channel activity as a function of time due to the presence of IVM, we measured the number of opening events per minute during the first 5 min for each condition (Figure 4D). In the control, the number of events remains constant during the course of the recording (Fig. 4D and [19]). However, in the presence of IVM, the frequency of events profoundly decreases after the first minute. This is clearly illustrated by the percentage of events detected in the first and second minute, which decreases from ∼80% to ∼10%, respectively.

The few opening events from IVM-treated cells were analyzed to determine if the drug affects single-channel properties. Open time histograms are fitted by the sum of two exponential components, which are identical to those detected in the control condition (Fig. 4E and Table 1). Closed-time distributions of L-AChRs from both conditions are well described by the sum of three or four components. The main closed component is displaced to longer durations in recordings from cells previously treated with IVM (*p*<0.01; Fig. 4E and Table 1), in agreement with the decreased frequency of opening events.

Taken together, our single-channel study reveals that muscle L-AChR and GABA receptors are profoundly inhibited by IVM.

### Behavioral effects of anthelmintic drugs

**Figure 5:**
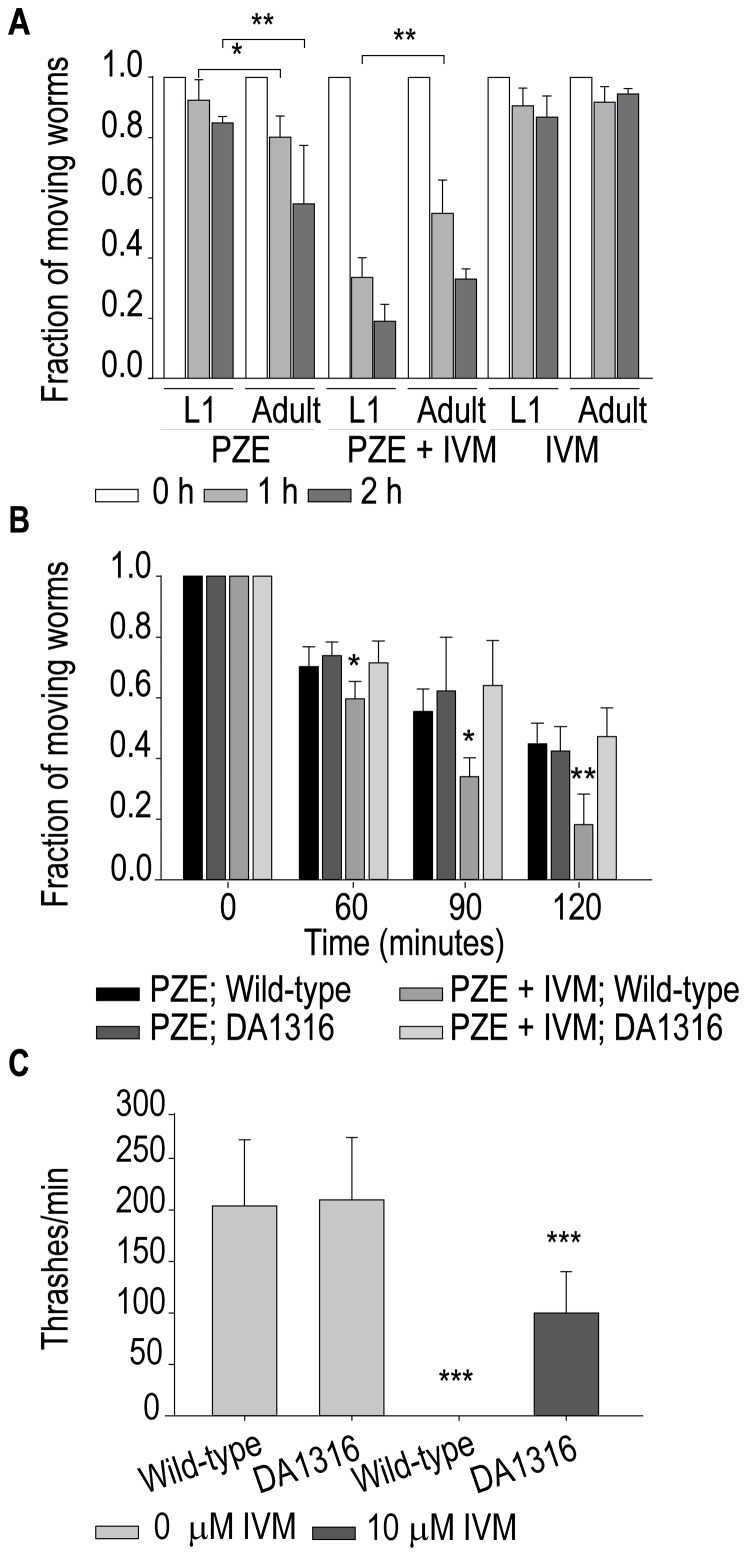
Measurement of sensitivity of *C. elegans* to PZE and IVM. (**A**) Synchronized L1 and adult wild-type worms were placed on plates containing the specified drug [25 mM Piperazine (PZE), and/or 300 µM ivermectin (IVM)], and observed at the indicated times to determine the fraction of worms that respond to prodding, which were considered as “moving worms”. (**B**) Wild-type and triple mutant *avr-14(ad1302);avr-15(ad1051);glc-1(pk54)* (DA1316 strain), adult worms were placed on agar plates containing the specified drug [40 mM PZE, and/or 300 µM IVM]. Each point represents the average of 3-5 experiments, n = 30 worms, error bars  =  SD. (**C**) Effect of IVM on nematode swimming measured as thrashes per minute for wild type and the triple mutant. Measurements were performed after 30 min of incubation in M9 buffer or M9 buffer plus 10 µM IVM.

PZE produces flaccid paralysis and lengthening of parasitic and *C. elegans* worms [5], [28]. Since our results show that PZE is an agonist of *C. elegans* UNC-49 channels in L1 cells, we also explored drug sensitivity at this stage. 2 h-exposure of worms to 1-10 mM PZE does not affect significantly worm locomotion whereas 25 mM PZE significantly affects worm behavior producing flaccid paralysis. Under this condition, ∼40% adult and ∼20% L1 worms are paralyzed after 2 h (Fig. 5A). The reduced sensitivity of L1 stage may be due to the fact that at this stage GABA receptors are not present in dorsal muscle and are only present in ventral muscle [29]. At 40 mM PZE ∼90% of L1 worms are paralyzed after 2 h on agar plates.

The features of IVM-induced paralysis have been well described [30], [31]. After 2-h exposure on agar plates containing 0.3 mM IVM, worms are stationary but they still respond to prodding (Fig. 5A). The combination of 0.3 mM IVM with 25 mM PZE significantly enhances the flaccid paralysis produced by 25 mM PZE alone, resulting in ∼75% of adult and ∼80% of L1 worms that do not respond to prodding after 2 h (Fig. 5A). Thus, IVM causes a synergic effect on PZE-induced paralysis at both stages. As a control, we verified that no changes in worm behavior occur in the presence of 1% DMSO alone. The PZE and IVM doses used in both human and veterinary medicine appear to be high (e.g. 50–110 mg/kg host weight for PZE and 0.2 –200 mg/kg host weight for IVM [32]–[37]), in close agreement with the concentrations required to show effects in our assays.

To gain further insights into IVM targets, we used the triple mutant strain on GluClRs subunits, *avr-14(ad1302);avr-15(ad1051);glc-1(pk54)*, which has been shown to exhibit high resistance to IVM [31]. Non statistical differences in the sensitivity to 40 mM PZE on agar plates are detected between wild-type and triple mutant worms (Fig 5B). In contrast, PZE-induced paralysis is enhanced by the presence of 0.3 mM IVM in wild-type, as shown above, but not in triple mutant worms (Fig. 5B), thus suggesting that GluClRs are involved in the IVM potentiation of PZE paralysis.

Thrashing assays in the presence of 10 µM IVM show that 100% of wild-type worms are stationary. The triple mutant worms show a reduction of 50% in the thrashing rate (Fig. 5C), suggesting that other receptors besides GluClRs may contribute to the IVM effect, at the micromolar range, on worm locomotion [31].

## Discussion

The prevalence of human and animal helminth infections remains staggeringly high, thus urging the need for concerted efforts towards this area of research [38], [39]. GABA receptors, encoded by the *unc-49* gene, mediate body muscle inhibition during locomotion in *C. elegans* and parasitic nematodes and are therefore important targets of anthelmintic drugs [2], [12], [13], [16]. Thus, the molecular and pharmacological characterization of these nematode receptors will have important implications for the development of novel anthelmintic drugs as well as for our understanding of GABA receptor pharmacology.

We here identified functional muscle GABA receptors composed of UNC-49 subunits in *C. elegans* L1 muscle cells. Not all L1 muscle cells do respond to GABA application, nor do they show GFP-label associated to GABA receptors, in agreement with the fact that at the L1 stage GABA receptors are expressed only in body ventral muscles and not in body dorsal muscles [29], [40]. Thus, our study gives further evidence that the L1 cell culture reproduces the *in vivo* situation [21].

Overall responses of GABA receptors to anthelmintic drugs were evaluated by measuring macroscopic currents from L1 muscle cells in the whole-cell configuration. Unfortunately, these cultured muscle cells are not technically suitable for successive drug applications and they therefore do not allow complete pharmacological assays. This relatively limited information is nevertheless still valuable when complemented with single-channel recordings and behavioral assays.

Macroscopic currents elicited by GABA show rapid onset as well as rapid and full decay under the sustained pulse of agonist, indicating full desensitization [19]. At 1 mM, muscimol elicits larger currents than those elicited by 1 mM GABA, indicating that it is a full and potent agonist of *C. elegans* UNC-49 receptors as of vertebrate GABA_A_ receptors. In contrast, muscimol has been shown to be less potent than GABA at *Ascaris suum* and *Haemonchus contortus* receptors [12], [41]. PZE is used to treat *Ascaris lumbricoides* and *Enterobius vermicularis* infections in humans. Nematode paralysis has been shown to be mediated by activation of GABA-currents in *A. suum* [42]. We here show that it is also an agonist of *C. elegans* muscle GABA receptors since it is capable of eliciting macroscopic currents, which are of reduced amplitude when compared to GABA-elicited currents.

*C. elegans* GABA currents have been recorded previously from oocytes expressing either UNC-49B/C or UNC-49B subunits [43]. The GABA-activated currents recorded from our L1 cultured cells seem to be more similar to those corresponding to UNC-49B/C heteromers than to UNC-49B homomers, which show significantly slower desensitization [43].

In spite of the low number of active patches and the few opening events, we were able to describe the properties of single UNC-49 channels from L1 muscle cells. Channel openings activated by GABA, muscimol or PZE exhibit single amplitudes of ∼2.5 pA (+100 mV pipette potential) and brief open durations. The opening events are not detected in the absence of agonist or in patches from the *unc-49* null mutants, thus revealing that the observed channel activity is originated from UNC-49 receptors. The lowest PZE concentration that allows detectable opening events as well as the frequency of opening events is reduced when compared to the other agonists. These observations together with the reduced macroscopic currents suggest that PZE is less efficacious than GABA. In agreement with this conclusion, PZE has been shown to act as a low-efficacy agonist of GABA receptors of the parasitic nematodes *A. suum* [14], [42] and *H. contortus* [44].

Up to our knowledge the only -though limited- description of single *C. elegans* UNC-49 channel activity was made in HEK cells transfected with UNC-49B and C subunits [16]. The estimated conductance was ∼37 pS for UNC-49B homomers and ∼30 pS for UNC-49B/C heteromers [16].This latter value is in close agreement with the conductance determined from our single-channel recordings from L1 cells (∼22 pS) as well as with the conductance determined for GABA receptors from *A. suum* (∼22 pS, [45]). Thus, the comparison of the desensitization rate of macroscopic currents and the single-channel conductance determined in our study with results from heterologously expressed UNC-49 receptors suggests that receptors in L1 muscle cells may be UNC49B/C heteromers, as previously reported for both *C. elegans* and parasitic nematodes [9], [43], [44].

Although IVM has revolutionized the treatment of nematode and arthropod parasites, much remains to be learned about how it works and how resistant to it is developed [38]. The antiparasitic effects of IVM are known to be mediated by paralysis of the pharyngeal muscle through glutamate-gated chloride channel receptors (GluClR) [17], [46], [47] and of the somatic muscle through GABA receptors [48]. However, at the molecular level, IVM has been shown to activate and modulate a great number of Cys-loop receptors. IVM activates vertebrate GABA receptors, potentiates GABA receptors from *H. contortus* and inhibits GABA receptors from *A. suum* [5], [49], [50]. Our study reveals that it inhibits UNC-49 receptors from *C. elegans*. IVM inhibition appears to be irreversible (currents cannot be recovered after 5-min wash) in agreement with its reported actions at other anionic Cys-loop receptors [51]. IVM action at GABA receptors is more similar between *C. elegans* and the parasite *A. suum* than between the latter and *H. contortus,* in line with the fact that *C. elegans* is no more dissimilar to parasitic nematodes than each individual species of parasite is to another [17], [25].

Therefore, although GABA receptor subunit family appears conserved among nematodes there are variations in the properties of how individual channels respond to IVM among species. Understanding the molecular bases of these differences may lead to more rational and specific treatments.

With respect to AChRs, IVM has been shown to potentiate vertebrate α7 AChRs [52] and to inhibit *C. elegans* N-AChRs (ACR-16) [53] and α7-5HT_3_A chimeric receptors [54]. Our study extends the information of IVM targets by showing that it inhibits *C. elegans* L-AChR, which is the main excitatory receptor involved in locomotion. Assuming that the few detected openings from IVM-treated cells correspond to IVM-treated receptors, our detailed single-channel study reveals that IVM fully inhibits channel activity without affecting open-channel lifetime This observation may be mechanistically explained by the stabilization of desensitized states, inhibition of channel opening, or irreversible channel block. In conclusion, our study reveals that IVM inhibits *C. elegans* muscle GABA and AChR receptors, thus contributing to the understanding of the wide spectrum of action of this complex drug.

The crystal structure of GluClR complexed with IVM shows that the drug binds at subunit interfaces on the periphery of transmembrane domains between M3 on the principal subunit (+) and M1 on the complementary subunit (-) and it makes important contacts with the M2 pore-lining α-helix and the M2–M3 linker [55]. However, the relationship between binding and channel activation is not completely understood [56]. Based on the structure of GluCl-IVM and on how different receptors are activated or modulated by IVM, several residues in M2, M3 and M1 domains have been pointed as involved in activation and potentiation [51]. The comparison of these residues in UNC-49 subunits between *C. elegans* and *H. contortus* does not reveal a clear pattern of substitutions which may explain why their GABA receptors are oppositely modulated by IVM. Thus, this opposite IVM modulation could be due to different changes elicited by IVM binding rather than to IVM binding itself. In fact, mutations in residues from other loops outside the IVM-binding site affect the efficacy of IVM activation [54], [57]. By molecular docking and mutagenesis studies it has been suggested that the site of action of IVM at AChRs may be located at an intrasubunit cavity (not intersubunit as in the GluCl receptor) in the transmembrane region [54], [58]. Thus, the occupancy of different sites or/and the intrinsic capability of each receptor to respond to IVM binding may lead to the pleiotropic effects of this drug.

Understanding the mechanistic and structural bases underlying the final response of IVM may not only be important for the development of novel anthelmintic agents but also for drugs targeting human Cys-loop receptors associated to neurological diseases. In this respect, drugs that alter receptor responses to normal released neurotransmitter, such as positive allosteric modulators, are emerging as key therapeutic treatments for neurodegenerative and neurological diseases [59].

The combination of drugs is a strategy tending to fight against the increase in anthelmintic resistance. Despite being a low-efficacy GABAergic agonist, PZE is capable of inducing flaccid paralysis in *C. elegans*. We show that IVM can be used to potentiate the paralysis induced by PZE (at both L1 and adult stages) and that this potentiation may be mediated by GluClRs since it does not occur in the triple mutant strain. The explanation for the synergic effect seems to be not so straightforward since IVM inhibits UNC-49 receptors but potentiates PZE flaccid paralysis. However, IVM action is highly complex given its broad spectrum of action [48]. Although understanding the neuronal circuit basis underlying the synergic IVM-PZE effect requires further investigation, our results suggest that the combination of the two drugs may result in a good alternative strategy to overcome the ever increasing resistance of parasites to these drugs, which is of global concern for human and cattle health.
